# Genomic Insights into *Edwardsiella ictaluri*: Molecular Epidemiology and Antimicrobial Resistance in Striped Catfish (*Pangasianodon hypophthalmus*) Aquaculture in Vietnam

**DOI:** 10.3390/microorganisms12061182

**Published:** 2024-06-11

**Authors:** Vera Irene Erickson, Tu Thanh Dung, Le Minh Khoi, Yaovi Mahuton Gildas Hounmanou, Tran Minh Phu, Anders Dalsgaard

**Affiliations:** 1 Department of Veterinary and Animal Sciences, University of Copenhagen, 1870 Frederiksberg, Denmark; vera.erickson@sund.ku.dk (V.I.E.); gil@sund.ku.dk (Y.M.G.H.); 2 Department of Aquatic Pathology, Can Tho University, Can Tho 94000, Vietnam; ttdung@ctu.edu.vn (T.T.D.); lmkhoi@ctu.edu.vn (L.M.K.); 3 Department of Aquatic Product Processing, Can Tho University, Can Tho 94000, Vietnam; tmphu@ctu.edu.vn

**Keywords:** *Edwardsiella ictaluri*, bacillary necrosis (BNP), *Pangasianodon hypophthalmus*, striped catfish, *bla*_CTX-M-15_, aquaculture, genomic epidemiology

## Abstract

*Edwardsiella ictaluri* is responsible for causing bacillary necrosis (BNP) in striped catfish (*Pangasianodon hypophthalmus*) in Vietnam. This study offers a comprehensive genomic characterization of *E. ictaluri* to enhance understanding of the molecular epidemiology, virulence, and antimicrobial resistance. *E. ictaluri* isolates were collected from diseased striped catfish in the Mekong Delta. The species was confirmed through PCR. Antimicrobial susceptibility testing was conducted using minimum inhibitory concentrations for commonly used antimicrobials. Thirty representative isolates were selected for whole genome sequencing to delineate their genomic profiles and phylogeny. All strains belonged to ST-26 and exhibited genetic relatedness, differing by a maximum of 90 single nucleotide polymorphisms. Most isolates carried multiple antimicrobial resistance genes, with the *tet*(*A*) gene present in 63% and *floR* in 77% of the genomes. The ESBL gene, *bla*_CTX-M-15_, was identified in 30% of the genomes. Three plasmid replicon types were identified: IncA, p0111, and IncQ1. The genomes clustered into two clades based on their virulence gene profile, one group with the T3SS genes and one without. The genetic similarity among Vietnamese isolates suggests that disease spread occurs within the Mekong region, underscoring the importance of source tracking, reservoir identification, and implementation of necessary biosecurity measures to mitigate spread of BNP.

## 1. Introduction

*Edwardsiella ictaluri* is a Gram-negative intracellular bacterial pathogen belonging to the family *Hafniaceae* [1]. It was first discovered and described in diseased channel catfish (*Ictalurus punctatus*) in the United States in 1976 [2]. Retrospective studies have shown that *E. ictaluri* was present already a decade before the first official description of the species [3]. *E. ictaluri* causes disease in several different farmed freshwater fish species globally, including different catfish species like striped catfish (*Pangasianodon hypophthalmus*) [4]. In Vietnam, *E. ictaluri* was identified and described in diseased striped catfish for the first time in 2002, when it was associated with a disease previously called “bacillary necrosis” [5,6]. In channel catfish, the disease is called enteric septicemia of catfish while it is known as bacillary necrosis of Pangasius (BNP) in striped catfish. Other fish species, like Nile tilapia and zebrafish, can also be infected with *E. ictaluri* [7,8].

Vietnam is the world’s leading producer of striped catfish and fish fillets are exported to many countries [9]. With the gradual intensification of production, the disease burden has increased. The two main diseases causing significant economic losses in striped catfish aquaculture are BNP and motile *Aeromonas* septicemia (MAS) [10]. In Vietnam, BNP is often associated with the rainy season when the water temperature drops below 28 °C. The disease can occur at any production stage, but especially fingerlings and fish in the first three months of grow-out are vulnerable to infection [10,11]. The onset of disease is often seen as a rise in mortality and clinical symptoms include emaciation, swollen abdomen, and petechial hemorrhages [5]. At a late stage of the disease, necropsy shows white nodular lesions on the liver, kidneys, and spleen [5,12].

In a questionnaire survey, Vietnamese striped catfish farmers were asked how often they experienced disease on their farms. Seventy-five percent of the farmers experienced BNP outbreaks during each production cycle [10]. Experimental infections with *E. ictaluri* have shown mortality rates from 50% to as high as 100% [13]. Farmers in Vietnam typically treat BNP by administrating feed containing florfenicol or doxycycline, and many farmers also treat the pond water with iodine and other chemicals and terminate feeding when disease occurs [4,10]. There is currently one approved injectable vaccine in Vietnam, ALPHA JECT^®^ Panga 2(PHARMAQ, Overhalla, Norway), which is based on formalin-inactivated cultures of *E. ictaluri* in combination with two serotypes of *Aeromonas hydrophila* [14]. However, the vaccine seems to not be widely used by Vietnamese striped catfish farmers [15].

There is a knowledge gap about the epidemiology and pathogenicity of the specific sequence types and/or genotypes of *E. ictaluri* causing disease in striped catfish, not only in Vietnam, but also globally. There are few available assembled *E. ictaluri* complete genomes in the European Nucleotide Archive (ENA; 11 paired reads) and in the National Center of Biotechnology Information (NCBI; 41 genomes). Most of these isolates originate from North America and only a few sequenced isolates originate from Southeast Asia, including one isolate from Nile Tilapia in Vietnam. In this study, we isolated 66 *E. ictaluri* from diseased striped catfish in the Mekong Delta area in Vietnam. We selected 30 isolates for Illumina MiSeq whole genome sequencing (WGS). Our library analyses of 30 sequenced isolates of *E. ictaluri* provides, for the first time, comprehensive genomic characteristics of *E. ictaluri* causing BNP in Vietnamese striped catfish, including information about virulence factors, antimicrobial resistance genes, mobile genetic elements, the pangenome, and phylogeny.

## 2. Materials and Methods

### 2.1. Edwardsiella ictaluri Strain Collection

Between 2017 and 2021, 72 bacterial isolates were collected from farmed striped catfish (*P. hypophthalmus*) showing signs of BNP. The isolates were collected from commercial striped catfish farms located in the Mekong Delta provinces of Dong Thap, An Giang, Can Tho, Ben Tre, Vinh Long, Tien Giang, and Long An. The isolates were obtained from fingerlings (size 5–100 g) and fish in grow-out ponds (size 100–800 g). To aseptically obtain samples from the liver and kidneys, fish were euthanized using Aqui-S^®^ (Bayer, Ho Chi Minh City, Vietnam), and the skin was sanitized with 70% alcohol prior to sampling. Samples were sub-cultured on tryptic soya agar (TSA) plates (Merck, Darmstadt, Germany) and incubated at 28 °C for 48 h. White pinpoint colonies were re-streaked onto new TSA plates. The pinpoint colonies were characterized by microscopy to distinguish their morphology and by biochemical characterization with Gram staining, catalase, oxidase, and O/F tests. Seventy-two *E. ictaluri* isolates representing different provinces and dates of isolation were included for further analysis. Isolates were kept in tryptic soya broth (TSB, Merck, Germany) containing 25% glycerol and stored at −80 °C until further study.

### 2.2. Bacterial Species Confirmation by PCR

To confirm that isolates were *E. ictaluri*, we performed species-specific colony PCR with the following primers: Edi-F 5′ CAG ATG AGC GGA TTT CAC AG 3′ (forward read) and Edi-R 5′ CGC GCA ATT AAC ATA GAG CC 3′ (reverse read) as described previously [16]. The expected band size of the amplicon was 470 bp.

### 2.3. Antimicrobial Susceptibility Testing

Antimicrobial susceptibility testing was performed for isolates identified as *E. ictaluri* using PCR by investigating the minimum inhibitory concentration (MIC) for commonly used antimicrobials in striped catfish farming in Vietnam, including florfenicol and oxytetracycline. The MIC testing was performed using Sensititre™ Avian AVIAN1F AST plates (ThermoScientific^TM^, Waltham, MA, USA) following the manufacturer’s guidelines. The plates contained the following antimicrobials: enrofloxacin, gentamicin, ceftiofur, neomycin, erythromycin, oxytetracycline, tetracycline, amoxicillin, spectinomycin, sulphadimethoxine, trimethoprim/sulfamethoxazole, florfenicol, sulphathiazole, penicillin, streptomycin, novobiocin, tylosin tartrate, and clindamycin. *Escherichia coli* ATCC25922 was used as control. After incubation, the Sensititre plates were analyzed with the help of the Thermo Scientific™ Sensititre™ SWIN™ Software System. No breakpoints have been established to determine clinical susceptibility and resistance of *E. ictaluri* isolated from diseased fish. Therefore, we could only establish the MIC for the investigated antimicrobials.

### 2.4. Whole Genome Sequencing

From the collection of 72 isolates, we selected 30 isolates for whole genome sequencing. The criteria for selection included a positive PCR result for *E. ictaluri*, with further selection based on province origin and year of isolation. Genomic DNA extraction was performed using the bacterial DNA extraction kit Maxwell^®^ RSC Cultured Cells DNA Kit (Promega Corporation, Madison, WI, USA) following the manufacturer’s protocol. The DNA QC and Illumina sequencing were performed as previously described [17]. The sequencing process aimed to achieve a minimum sequence coverage of 50. Five isolates were chosen for Nanopore sequencing (EI08, EI16, EI17, EI41, and EI67) using Oxford Nanopore MinION sequencing (Oxford Nanopore Technologies, Oxford, UK) using the protocol SQK-RBK114.24 with the R.10 MinION flow cells. These five isolates were specifically chosen to provide a good representation of the entire set of 30 isolates, ensuring that as many of the characteristics and features as possible observed in these samples were covered.

### 2.5. Bioinformatic Analysis

The quality of the raw sequence was assessed using the quality assessment tool FastQC v0.11.9 [18]. To improve the quality of the raw sequences, trimming was performed with Trimmomatic version 0.3 [19] and the quality of the trimmed sequences was assessed using FastQC. Trimmed sequences were assembled with SPAdes 3.15.5 genome assembly tool [20]. The quality of the assembled genomes was assessed with QUAST version 5.2 [21]. The isolates sequenced using Nanopore MinION were basecalled and demultiplexed using Guppy v6.5.7 [22]. The obtained fastq reads were then filtered using filtlong with a minimum length threshold of 1000 bp. These long reads, together with their corresponding short reads from Illumina, were then subjected to hybrid assembly using Unicycler (https://github.com/rrwick/Unicycler, accessed on 2 May 2024). Species confirmation of the genomes was performed using Kraken2 [23] and KmerFinder version 3.2 [24,25,26].

Multi-locus sequence typing (MLST) was performed on the command line with MLST tool which relies on the PubMLST *Edwardsiella* spp. database [27]. The hybrid genomes served, in this analysis, to bait all 11 housekeeping genes of *Edwardsiella* to determine the exact MLST allelic matches from the PubMLST scheme. To screen the draft genomes for antimicrobial resistance genes, metal, and biocide resistance genes, we used the MEGARes database V3.0 [28] prompted with abricate v1.0.1 [29,30]. As there was no existing species-specific database for *E. ictaluri*, we created a database with relevant virulence genes based on previously published literature on virulence factors in *E. ictaluri* and screened the sequenced genomes for the presence of these genes. The database file is accessible as a text file in figshare through the public DOI: 10.6084/m9.figshare.25334749. Plasmid replicons were detected using the Enterobacterales database in PlasmidFinder 2.1 [31,32]. We used BLAST (https://blast.ncbi.nlm.nih.gov/Blast.cgi, accessed on 1 August 2023) to look for similar plasmid replicons matches in the NCBI database. Genomes were screened for mobile genetic elements with MobileElementFinder database v.1.0.2 [33], and for prophages using PHASTER [34,35]. We included all prophages that were either intact or of questionable completeness.

The genomes were annotated in Prokka version 1.14.5 [36] and the output annotation files in .gff format were used to perform a pangenome analysis in Roary version 3.13.0 [37]. The visualization of the pangenome was carried out using Phandango version 1.3.1 [38]. We conducted two separate pangenome analyses; one including only the *E. ictaluri* isolates sequenced in this study, and another analysis including all publicly available *E. ictaluri* isolates from NCBI (like the phylogenetic analysis). The pangenome analysis was constructed using the data from the Roary pangenome analysis visualized on the phylogenetic tree constructed using Snippy.

To put our strains in a global context, we retracted all *E. ictaluri* whole genomes present in Genbank for a comparative phylogenetic analysis. A search for all available *E. ictaluri* assembled genomes in FASTA format was conducted in the National Center for Biotechnology Information (NCBI) database, a search which generated 41 hits. For a genome to be included in the phylogenetic analysis, there had at least to be metadata available about the source of isolation. Based on this selection criterion, a total of 38 genomes were included in the phylogenetic analysis. If additional metadata, like the sampling date and geographical location, were available, these were recorded. All isolates were from fish from the United States (28 isolates), China (3), Japan (6), Thailand (1), Vietnam (1), and one isolate was of unknown origin. The reference genome of *E. ictaluri* ST-19 (ATCC 33202), isolated in 1976 from a channel catfish in the United States, was used for variant calling in the phylogenetic tree. The list of genomes and the available metadata can be found in Supplementary Table S1: NCBI sequence metadata. All genomes were then analyzed for single nucleotide polymorphisms where variants were labeled by using Snippy version 4.6.0 [39] under the following parameters: mapping quality = 60, minimum base quality = 13, minimum read coverage = 4, and 75% concordance at a locus. The *E. ictaluri* strain ATCC 33202 (GenBank accession: GCA_000264785.1) was used as a reference during variant calling and alignment. The core genome single-nucleotide variants were aligned with Snippy-core version 4.1.0 for phylogeny inference.

Putative recombinogenic regions were detected and masked using Gubbins version 2.4.1 [40]. A maximum-likelihood phylogenetic tree was build using RAxML version 8.2.12 and the generalized time-reversible model with 200 bootstraps [41]. The final tree was rooted on the reference genome and the tree was annotated and visualized with iTOL version 3 [42]. We constructed a single nucleotide polymorphism matrix to support the phylogenetic results (Supplementary Table S2: SNP matrix). Furthermore, we conducted a reference-independent computation of our samples where the 30 whole genomes were compared pairwise for Average Nucleotide Identity (ANI) testing using fastANI [43] and visualized the generated matrix using R version 4.3.2.

The raw sequence reads from this study have been submitted and are accessible through the European Nucleotide Archive (ENA) under the designated project number PRJEB73297.

## 3. Results

### 3.1. Species Identification and Whole Genome Sequencing

**Figure 1:**
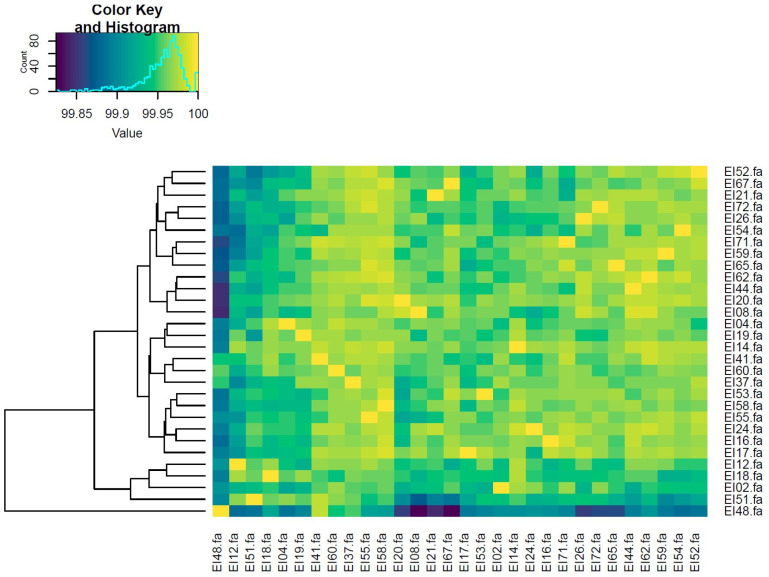
Pairwise average nucleotide identity between *E. ictaluri* genomes analyzed in this study. The genomes all had more 99% nucleotide identify varying from dark blue to bright yellow.

From the collection of isolates, PCR analysis showed the expected band size of 470 bp for 66 isolates and confirmed as *E. ictaluri*. Thirty representative *E. ictaluri* isolates were sequenced. The species *E. ictaluri* was confirmed from the WGS data for all isolates using Kmer-based identification in Kraken2. General information about the isolates is provided in Table 1. As expected, the size of the sequenced genomes ranges from 3.55–3.75 Mbp with a GC content range from 57.0–57.7%. All genomes were assigned to sequence type 26 (ST-26) based on the hybrid assemblies of long and short-reads. The 30 sequenced strains were isolated over 5 years (2017 to 2021) across different provinces of An Giang (4), Ben Tre (3), Can Tho (7), Dong Thap (11), Long An (1), Tien Giang (3), and Vinh Long (1) and showed a high overall similarity when compared against each other with an average nucleotide identity (ANI) of 99% and above (Figure 1).

### 3.2. Phylogenetic Analysis

**Figure 2:**
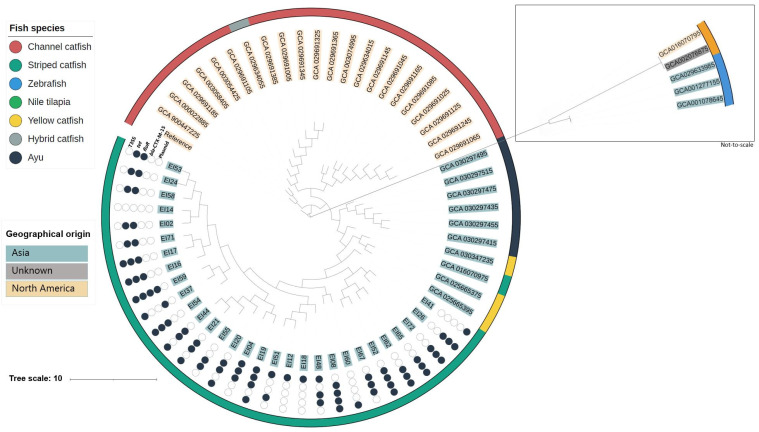
Maximum likelihood phylogenetic tree showing all publicly available *E. ictaluri* genomes from NCBI (40 isolates) and the 30 *E. ictaluri* isolates obtained from striped catfish in the Mekong Delta. The outer ring illustrates which fish species the isolate originates from. The colored text indicates which geographical origin the isolates come from (Asia, North America, unknown). Due to the big difference in SNPs, the branch with isolates from Nile Tilapia and zebrafish was added manually for the sake of the visualization. This branch is not-to-scale with the rest of the phylogenetic tree. The circles present the presence (black circle) or absence (empty circle) of the following parameters: T3SS, *tet* tetracycline resistance gene, *floR* florfenicol resistance gene, *bla*_-CTX-M-15_ beta lactamase, and plasmids.

In a global context, the phylogenetic analysis (Figure 2) shows that the isolates from North America form one homogenous group that is very similar to the reference genome, while the isolates from Asia group closely together. The exceptions are three isolates from zebrafish originating from the United States and two isolates (one from Nile Tilapia in Thailand and one from striped catfish with unknown origin), which form their own clade. As already shown by the ANI analysis, the phylogenetic analysis against a reference confirmed that the genomes of *E. ictaluri* isolated from striped catfish in Vietnam were closely related and were a maximum of 90 SNPs apart from each other. The isolates from Vietnam differed with 90–120 SNPs to the *E. ictaluri* isolated from Ayu fish in Japan and by 100–120 SNPs to isolates from yellow catfish in China. The difference between the isolates from Vietnam and the isolates from North America (United States) was >500 SNPs. The difference in SNPs was > 6000 between the isolates from Nile tilapia and the isolates from striped catfish in Vietnam. These findings point to two potential drivers in the molecular epidemiology of *E. ictaluri:* niche-based clonality and geographic dissemination patterns. The SNP matrix is available in Supplementary Table S2: SNP matrix.

Like the isolates from striped catfish in Vietnam, isolates from yellow catfish in China and Thailand belonged to ST-26. Despite the difference in SNPs, one of the isolates from Nile tilapia (GCA016070795) belonged to ST-26 while the other *E. ictaluri* isolate from Nile tilapia (GCA002076875) had ST-24. All the isolates from North America were either ST-19 or ST-23, or a match for both these sequence types.

### 3.3. Pangenome Analysis

**Figure 3:**
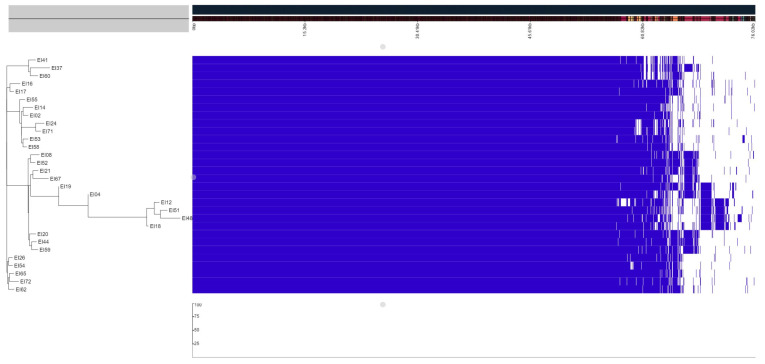
Pangenome of 30 *E. ictaluri* isolates associated with disease in striped catfish in Vietnam. The area marked as A shows the “virulence prone subgroup”, B is the “HGT subgroup”, and C and D are the “MGE prone subgroups”.

The pangenome for *E. ictaluri* isolates from Vietnam consisted of 2834 core genes and 937 accessory genes. Most of the accessory genes (569 genes) were hypothetical proteins. As seen in Figure 3, there are coding sequences that are present in some genomes, but not in all. We looked at the individual coding sequences to see what the function of these genes were. The coding sequences marked as “A” in Figure 3 consisted of the following genes: *mltC* (murein-degrading enzyme), *yscU* (T3SS protein), *spaQ* (likely related to T3SS), *spaP*, *ssaV* (T3SS function), *ssaN* (T3SS function) [44], *sseB* (T3SS) [45], *sctC* (T3SS secretin), *rhaS* (L-rhamnose operon regulatory protein), *guaD* (guanine deaminase), *yedY* (protein-methionine-sulfoxide reductase catalytic subunit), and *yedZ* (protein-methionine-sulfoxide reductase heme-binding subunit). Based on the type of genes found in group A, which were dominantly virulence related genes, this group was labelled as the “virulence prone subgroup”. The coding sequences marked “B” contain various tra genes (conjugal transfer proteins); *traA*, *traV*, *traC*, *traN*, *traQ*, *traS*, *traD*, *traI*, *traM*, *finO* (conjugal transfer repressor), *psiB* (plasmid SOS inhibition protein B), *noc* (nucleoid occlusion protein), *ssb*, *klcA* (antirestriction protein), *yhdJ* (methyltransferase), *umuD* (mutagenic process), *umuC* (mutagenic process), *stbB* (plasmid stability protein), *parM* (plasmid segregation protein), and *xerC* (tyrosine recombinase). Isolates in this category were regarded as harboring more plasmid-encoded genes and may be more likely to be involved in horizontal gene transfer (HGT) events. Group B was therefore labelled as the “HGT subgroup”. Coding sequences in “C” were: *soj*, *ftsH* (ATP-dependent zinc metalloprotease), *addA* (adenosine deaminase), *hns* (DNA-binding protein), *tus* (DNA replication terminus site-binding protein), *yraJ* (outer membrane protein), *faeE* (chaperone protein), *faeG* (chaperone protein), *pdeL* (phosphodiesterase), *emrE* (ethidium bromide-methyl viologen resistance protein), IS6 transposase, IS66 transposase, and IS481 transposase. The last area marked as “D” consisted of the following unique genes: *dsbC* (Thiol:disulfide interchange protein), *traC* (conjugal transfer proteins), *pdeG* (phosphodiesterase), *cobS* (adenosylcobinamide-GDP ribazoletransferase), repB (replication protein), and IS91 transposase. Many of the genes in group C and D were related to mobile genetic elements (MGEs). Both the C and D groups are “MGE prone subgroups” as they seem to contain distinct coding sequences encoding for mobile genetic elements supporting the HGT subgroup.

We further performed a pangenome analysis with the global strain collection to assess whether there were any apparent differences between the pangenome of the Vietnamese isolates and the isolates from other countries. The pangenome of all available *E. ictaluri* genomes consisted of 1907 core genes and 5718 accessory genes. Of the accessory genes, 2690 were hypothetical proteins. The genes present in all isolates from Vietnam, but absent in all other isolates, revealed that the majority of these were encoding hypothetical proteins. The Vietnamese isolates also contained the following unique genes: *cobS* (Adenosylcobinamide-GDP ribazoletransferase), *flhD*, *dsbC* (thiol:disulfide interchange protein), *flhC* (flagellar transcriptional regulator), *umuD*, *umuC*, *traC*, and *polC* (DNA polymerase III).

### 3.4. Antimicrobial Resistance Genes and Minimum Inhibitory Concentrations

**Figure 4:**
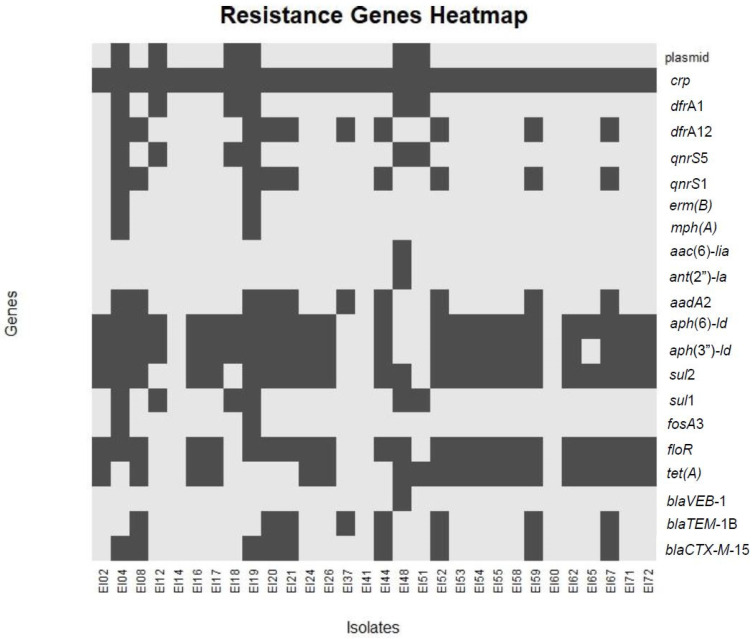
Antimicrobial resistance genes. Heatmap displaying the presence (dark grey color) or absence (light grey color) of the antimicrobial resistance genes.

The MIC was determined for all 66 isolates identified as *E. ictaluri* by PCR. The presence of antimicrobial resistance genes are reported only for isolates subjected to WGS. Most of the genomes of the 30 sequenced *E. ictaluri* isolates from Vietnam carried several antimicrobial resistance genes. All isolates (100%) carried the *crp* gene, a drug and biocide RND efflux regulator. The streptomycin resistance genes, *aph*(6)*-Id* and *aph*(3″)*-Ib*, were the most predominant antimicrobial resistance genes and were present in 80% and 77% of the genomes, respectively. The sulfonamide resistance genes, *sul*1 and *sul*2, were found in 20% and 77% of the genomes, respectively. The antimicrobial resistance gene *floR*, encoding resistance to florfenicol, was found in 77% of the genomes, and the tetracycline resistance gene *tetA* was found in 63% of the genomes. Less prevalent genes were *aadA*2 (33%), *qnrS*1 (30%), *qnrS*5 (20%), *dfrA*12 (33%), *dfrA*1 (20%), *fosA*3 (7%), *mph*(*A*) (7%), *erm*(*B*) (7%), *ant*(2″)*-Ia* (3%), and *aac*(6′)*-Iia* (3%). We also found the critical cephalosporin beta-lactamase encoding gene *bla*_CTX-M-15_ in 9/30 (30%) of the isolates. Furthermore, we found two other beta-lactamase genes; *bla*_TEM-1B_ (27%) and *bla*_VEB-1_ (3%). Three genomes (isolates EI14, EI41, and EI60) did not carry any antimicrobial resistance genes at all. On average, the isolates carried resistance genes towards four different drug classes of antimicrobials. Two isolates carried antimicrobial resistance genes towards seven different antimicrobial classes. The results from the antimicrobial resistance gene analysis can be seen in Figure 4.

**Figure 5:**
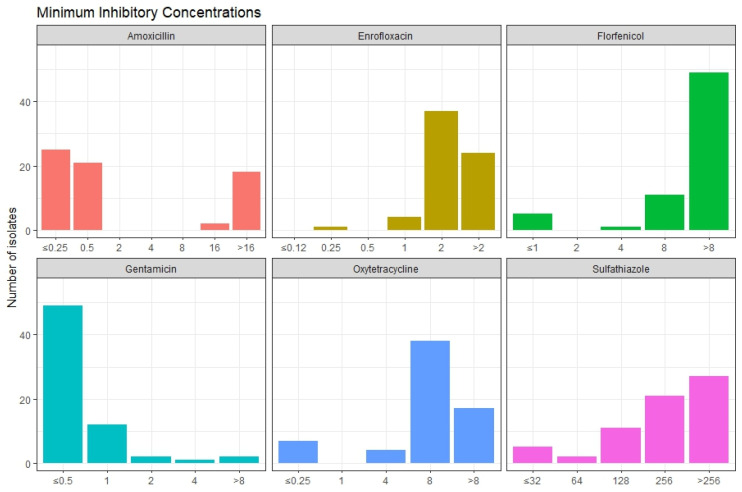
The minimum inhibitory concentration (MIC) for six different antimicrobials tested on 66 *E. ictaluri* isolates. The MIC value is displayed on the x-axis and the number of isolates is displayed on the y-axis.

The MIC of commonly used antimicrobials, such as florfenicol and oxytetracycline, was determined for all 66 isolates identified as *E. ictaluri* by PCR, including the 30 isolates selected for WGS. As there are no established breakpoints for determining antimicrobial susceptibility for *E. ictaluri* in aquaculture, we used the epidemiological cutoff values to determine susceptibility and resistance when possible. We tested the MIC of our *E. ictaluri* isolates, and the MIC results for six antimicrobials are visualized in Figure 5. Forty-nine isolates had an MIC ≥ 8 mg/mL for florfenicol and 23/30 of the sequenced isolates carried the florfenicol resistance gene *floR*. None of the isolates with an MIC < 8 mg/mL for florfenicol carried the *floR* resistance gene. For oxytetracycline, 11 isolates had an MIC < 8 mg/mL, while the remaining 55 isolates had an MIC ≥ 8 mg/mL. Two of the sequenced isolates with MIC < 8 mg/mL for oxytetracycline carried the *tet*(*A*) tetracycline resistance gene. Forty-six isolates had an MIC of ≤0.5 mg/mL for amoxicillin, while twenty isolates had an MIC ≥ 16 mg/mL. Only the isolates with MIC ≥ 16 mg/mL for amoxicillin carried the *bla*_CTX-M-15_ beta-lactam resistance gene. However, we did not include any cephalosporin antimicrobials in the MIC testing. Sulphathiazole showed MIC ≤ 128 mg/mL in 18 isolates. Out of the sequenced isolates with MIC ≤ 128 mg/mL for sulphathiazole, five isolates carried the sulfonamide resistance gene *sul*2. The rest of the isolates had an MIC ≥ 256 mg/mL for sulphathiazole and 19 of the sequenced isolates with MIC ≥ 256 mg/mL carried the *sul*2 gene. The MIC of enrofloxacin was ≤2 mg/mL in 42 isolates, and >2 mg/mL for the remaining 24 isolates.

### 3.5. Virulence Genes

We screened the genomes against our developed database containing 69 putative *E. ictaluri* virulence genes. From the results, it was clear that the genomes grouped into two different virulence profiles. The first group, containing 13 genomes (13/30), did not have any gene matches for any of the proteins in the type III secretion system (*esaL*, *esaK*, *esaJ*, *esaI*, *esaH*, *esaG*, *esrC*, *esaD*, *esaC*, *esaB*, *eseG*, *escB*, *eseE*, *eseD*, *escC*, *escA*, *eseB*, *eseA*, *esaQ*, *esaP*, *esaO*, *esaN*, *esaV*, *esaM*, *esaR*, *esaS*, *esaT*, *esaU*, *esrA*, and *esrB*), while the second group of 17 genomes (17/30) had matches for all of these genes involved in the type III secretion system. The *virD*4 gene, a putative T4SS gene, was not found in any of the genomes. One genome, EI54, differed slightly as the only genome carrying the putative virulence genes *mobA*, *mobB*, and *mobC*. These three genes are molybdenum cofactor biosynthesis proteins. The *TolQ*, *TolR*, *TolA*, and *TolB* genes were present in all genomes. These four envelope proteins have been suggested to contribute to *E. ictaluri* virulence. We screened the genomes for nine genes known to play a part in *E. ictaluri* urease activity (*ureA*-*ureG*, *ureI* and *amtB*). All nine genes were present in all thirty (30/30) genomes. The virulence gene database is available as a text file in figshare through the public DOI: 10.6084/m9.figshare.25334749.

### 3.6. Plasmids, Mobile Genetic Elements and Prophages

Six of the isolates carried plasmid replicons in their genomes: EI04, EI12, EI18, EI19, EI48, and EI51. The plasmids IncA/C2, InQ1, and p0111 were detected and isolate EI48 carried all three plasmids. The isolates EI04 and EI19 carried the beta-lactamase gene *bla*_CTX-M-15_ and EI48 carried *bla*_VEB-1_. The isolates carrying plasmids also carried the *qnrS*5 quinolone resistance gene and the trimethoprim resistance gene *dfrA*1. No other isolates carried these two genes. Summary information about the plasmids is presented in Table 2. The conjugative plasmid IncA/C found in six isolates had a 100% identity and 95–100% query coverage with the plasmid replicon pEI-2234-3 (accession number: CP053782, unpublished work) originating from an *E. ictaluri* isolate from Thai Binh province in Vietnam (isolation source unknown). The IncA/C plasmid replicon also had a 90% identity with a plasmid isolated from a bacterial strain of *Providencia rettgeri* (accession number: LC507075) isolated from human urine in Japan [46]. The p0111 plasmid replicons from the Vietnamese *E. ictaluri* isolates had a 99.94% identity and 80–89% query coverage with an *E. coli* plasmid replicon p16EC-p0111 isolated from a pig in China (accession number: MN086777.1) and another *E. coli* plasmid replicon pJSMCR1_2 isolated from chicken in South Korea (accession number: CP030154.1). The plasmid replicon p0111 from the Vietnamese isolate EI48 was highly similar (99.60% identity and 100% query coverage) to the *E. coli* plasmid replicon pS142-1 isolated from broiler chicken in France (accession number: OY754437.1). The p0111 plasmid replicon from the isolate EI51 was most similar to the *E. coli* plasmid replicon pMRE162 UK (99.92% identity and 99% query coverage), isolated from human feces in the United Kingdom (accession number: CP119405.1). The plasmid replicon IncQ1 from the Vietnamese isolate EI48 was similar to two plasmid replicons from two different *Vibrio* spp. (accession numbers KX539265.1 and KX575838.1), both isolated from shrimp in China, and an *E. coli* plasmid replicon (accession number: CP119405.1) isolated from human feces in Lao PDR.

Each of the thirty isolates also carried at least one prophage. Nine different prophages were identified using PHASTER. Some of the isolates (6/30) carried two or more prophages (details are presented in Supplementary Table S3: Prophage results). All sequenced isolates carried at least one type of mobile genetic element, either insertion sequences or composite transposons. Ten different types of insertion sequences or transposons were found. The composite transposon ISVsa3 was found in 15/30 isolates. The three most common insertion sequences found were ISEcl10 (28/30), ISVsa3 (23/30), and IS630 (18/39). A summary of the analysis for mobile genetic elements is provided in Supplementary Table S4: Mobile Genetic Elements Results.

## 4. Discussion

We analyzed genomes of 30 *E. ictaluri* isolated from diseased striped catfish over a five-year period from seven different provinces in the Mekong Delta. Despite the time span and the geographically dispersed origin of the samples, the strains were all the same sequence type, ST-26, and were genetically closely related. Thus, one common sequence type seems associated with bacillary necrosis in Pangasius (BNP) in the Mekong Delta. However, based on the pangenome and virulence gene analyses, we observed differences in gene profiles within ST-26. Based on the virulence gene profile, the isolates can be divided into two different groups, one with genes coding for the T3SS and another group with strains without the T3SS. Based on the pangenome analysis (Figure 3), we identified three different gene profiles. We suggest that the different gene profiles identified in this study are considered when developing vaccines to maximize the effectiveness of vaccines. The current commercially available vaccine for prevention of BNP contains one single *E. ictaluri* strain [14] and it remains to be documented to what extent this vaccine provides adequate immune response and protection against infections caused by strains with different gene profiles as reported in our study. Striped catfish farmers in Vietnam are generally quite hesitant to vaccinate their fish for a number of reasons [15]. A major practical and logistical challenge is the administration of the vaccine per intra-peritoneal injection. There is clearly a need to automatize the vaccination like is done in the salmon industry, but also to increase efforts to develop an immersion vaccine that protects against the different genetic types of *E. ictaluri* associated with BNP.

The phylogenetic analyses indicate that our strains are genetically distinct from those originating in other countries (Figure 2). This suggests that outbreaks of BNP in Vietnamese striped catfish are not likely attributable to the introduction of new clades, but rather to inadequate biosecurity measures at individual farms. Thus, there is a need to identify main sources and transmission routes of *E. ictaluri*. Because of the similarity of the isolates, source tracking could also be an effective method of managing the spread of disease on farms and between farms. A previous investigation suggested that the transmission of *E. ictaluri* is influenced by the structure of the striped catfish farming system in the Mekong Delta [47]. Farms often share water from the Mekong River canal system, and many companies operate vertically integrated production systems. This scenario could facilitate the spread of isolates across provinces, for example, through the transport of fingerlings [47]. *E. ictaluri* may disseminate to different farms via shared resources such as equipment, fingerlings, and water sources. Overall, our findings suggest that controlling BNP may be feasible through comprehensive risk analysis and source tracking of the bacterial pathogen. However, there is limited knowledge about reservoirs of *E. ictaluri* and their potential role in spreading the pathogen to different farms.

The evidence provided by this study suggests that the *E. ictaluri* strains causing disease at Vietnamese striped catfish farms demonstrate a geographic or country-specific occurrence and spread, indicating environmental adaptation mechanisms that facilitate their persistence in such aquatic environments. These mechanisms may include the formation of biofilm and the ability of *E. ictaluri* to adhere to particles, including living organisms in water. Thus, studies are needed to determine if *E. ictaluri* is present in pond water and sludge as well as in nearby water bodies surrounding striped catfish farms in Mekong Delta. Other studies have suggested that biofilm may serve as a potential reservoir and play a role in the ecology of *E. ictaluri* [48,49], but more work is needed to confirm this.

Although genetically related, the accessory genome of the Vietnamese *E. ictaluri* isolates displayed microevolution by forming different clades, allowing us to distinguish strains that are more “virulence-prone” from those that are more “mobile genetic element-prone” from their unique composition in mobile elements related coding sequences. Notably, the clade comprising isolates EI12, EI18, EI48, and EI51 exhibited genomes that differed by only 40–59 SNPs between each other but showed the greatest variation compared to other genomes. Interestingly, there was no apparent correlation between geographical origin, isolation year, and the clustering of isolates in specific clades; these isolates were sourced from three different provinces (Can Tho, Dong Thap, and An Giang) and were isolated in different years (2017, 2018, 2019, and 2020). This observed microevolution in the genomes warrants further investigation. Genes within the region marked as “D” were unique to isolates EI12, EI18, EI48, and EI51. These genes included *dsbC*, involved in disulfide bond formation between proteins [50]; *traC*, encoding a gene coding a conjugal transfer protein essential to plasmid conjugation [51]; *pdeG* (phosphodiesterase); *cobS* (adenosylcobinamide-GDP ribazoletransferase); *repB* (replication protein); and IS91 transposase. These genes are all associated with mobile genetic elements. Further studies are needed to determine the significance of these groups in the pathogenicity and spread of the different strains.

Several of our genomes harbored critical resistance genes such as *bla*_CTX-M-15_ along with the presence of conjugative plasmids like IncA/C. The detection of extended-spectrum beta-lactamases (ESBLs), like *bla*_CTX-M-15_, in bacterial fish pathogens is uncommon and necessitates further monitoring of ESBLs and their associated mobile elements in fish farming environments. Previously, CTX-resistant *E. coli* has been identified in 61% of pond-reared striped catfish in the Mekong Delta [52]. Another study found that a 14.6% prevalence of ESBL-resistant *E. coli* in freshwater fish sold in wet markets in the Mekong Delta [53]. Further studies should determine possible transfer of the IncA/C and associated resistance genes between *E. ictaluri* and *E. coli*.

One of the genomes, EI48, harbored an IncQ1 plasmid. Three antimicrobial resistance genes were detected on the same node as this plasmid; *aac*(6′)*-Iia* (aminoglycoside resistance), *ant*(2″)*-Ia* (aminoglycoside resistance) and *bla*_VEB-1_ (beta-lactam resistance). We performed a BLAST search and found that the IncQ1 plasmid replicon closely matched plasmids isolated from shrimp farms in China and from a plasmid isolated from human feces in Lao PDR. These IncQ1plasmids have also been found in *Vibrio* spp. and in *E. coli*, suggesting that this plasmid type can contribute to the spread of beta-lactam resistance across bacterial species and different host species. We obtained the plasmid sequences of these three closely matching IncQ plasmids (>98% similarity) and screened them for antimicrobial resistance genes. Similar to the IncQ1 plasmid replicon found in EI48, two plasmids from *Vibrio* spp. (accession numbers KX539265.1 and KX575838.1) and one *E. coli* plasmid (CP119405.1) from human feces also carried the *aac*(6′)*-Iia*, *ant*(2″)*-Ia*, and *bla*_VEB-1_ genes. Thus, it seems that plasmid replicons in our isolates closely matched plasmids in bacterial pathogens from different sources in East Asia and Southeast Asia, including livestock, poultry, and human feces. However, plasmid types between *E. ictaluri* isolated from catfish in Vietnam and the United States (typically plasmid profiles similar to pEI1 and pEI2, GenBank accession AF244083 and AF244084, respectively) seem different [54,55].

Most of the isolates carried antimicrobial resistance genes and exhibited phenotypic resistance to the key antimicrobials commonly used in the treatment of BNP, namely florfenicol and oxytetracycline. Most isolates showed MIC values of ≥8 mg/L for both oxytetracycline and florfenicol. Additionally, some isolates displayed low MIC values for ampicillin and penicillin. Understanding the antimicrobial susceptibility patterns of *E. ictaluri* and other pathogens causing disease on specific farms will enable farmers to select appropriate antimicrobials for treatment.

A significant challenge faced by veterinarians and farmers is the lack of treatment guidelines based on clinical breakpoints for antimicrobials. Currently, there are no established antimicrobial susceptibility breakpoints or epidemiological cut-off (ECOFF) values for *E. ictaluri* in neither EUCAST nor CLSI, and the interpretation of susceptibility to a given antimicrobial largely relies on the observed effect of the antimicrobial treatment and the veterinarian or farmer’s empirical experience [56]. In 2020, CLSI released the 3rd edition of the VET04 “Performance Standards for Antimicrobial Susceptibility Testing of Bacteria Isolated From Aquatic Animals” [57]. However, this guide includes only a few aquatic bacterial pathogens, and none of the pathogenic *Edwardsiella* spp. are included. The first step towards creating a treatment guideline for *E. ictaluri* associated with BNP is to establish the ECOFF values for relevant antimicrobials, like florfenicol and oxytetracycline, and collect evidence for the effect of these antimicrobials treating BNP infections in striped catfish. Individual researchers can contribute to the establishment of ECOFF values in EUCAST by submitting their results from MIC or disk diffusion testing of antimicrobials when the bacterial pathogen has been identified at species level, two-fold MIC dilutions were used, and the method preferably followed the ISO 20776-1:2019 standard [58]. Establishing ECOFFs is the first step towards clinical breakpoints for susceptibility and resistance for antimicrobials approved for use in aquaculture. There is an urgent need to establish such treatment guidelines for *E. ictaluri* infections in Vietnamese striped catfish.

Our understanding of the functions of genes in *E. ictaluri* remains limited, with no available database covering virulence genes specific to *Edwardsiella* species. Consequently, the importance of putative virulence genes or virulence factors in *E. ictaluri* is largely unknown, and existing knowledge is primarily derived from studies of *Edwardsiella tarda*, a bacterial pathogen known to infect aquatic species and humans [59]. Traditionally, studies have focused on investigating individual putative virulence genes rather than comprehensive virulence factors. To address this gap, we have developed a new database comprising genes encoding several virulence factors, including the T3SS system, as well as individual putative virulence genes specific to *E. ictaluri*. This database contains the amino acid sequences of 69 putative virulence genes and is readily accessible for download as a text file from figshare (public DOI: 10.6084/m9.figshare.25334749). Researchers can utilize this database to screen *E. ictaluri* genomes for putative virulence factors, enhancing our understanding of the pathogenicity mechanisms of this bacterium.

Moving forward, it is imperative that future studies validate the virulence of these putative virulence factors and genes. This can be achieved through infection experiments involving striped catfish with knockout mutants of *E. ictaluri*, among other approaches. Upon confirmation of putative virulence genes, efforts can be directed towards establishing a comprehensive database for screening virulence genes in *Edwardsiella* species, thereby advancing our knowledge of the pathogenic mechanisms of these bacteria.

## 5. Conclusions

The genomic data obtained from the analysis of 30 isolates in this study offers valuable insights into the recent strains of *E. ictaluri* circulating within the striped catfish (*Pangasianodon hypophthalmus*) production in the Mekong Delta, Vietnam. Notably, sequence type ST-26 was identified as the predominant strain associated with all outbreaks of bacillary necrosis of Pangasius (BNP) in this region, indicating a country-specific dissemination of identical clones. This underscores the importance of source tracking and reservoir identification to enhance the implementation of necessary biosecurity measures on farms.

Furthermore, a significant proportion of the isolates carried antimicrobial resistance genes, including those clinically relevant to human health, such as the ESBL *bla*_CTX-M-15_. The genomes also harbored plasmid replicons capable of facilitating the spread of antimicrobial resistance genes. Given these findings, it is imperative to strengthen biosecurity measures and explore alternative preventive strategies, such as vaccinations, to effectively mitigate future outbreaks of BNP.

Additionally, the establishment of antimicrobial susceptibility breakpoints for relevant aquatic pathogens, including *Edwardsiella* spp., is critical for guiding more targeted and judicious antimicrobial use in aquaculture. This proactive measure can help reduce overall antimicrobial usage and minimize the emergence and dissemination of antimicrobial resistance in aquatic environments, in foods and ultimately to humans.

## Figures and Tables

**Table 1:** General information about the 30 sequenced *E. ictaluri* isolates. PCR: result from species identification using PCR; Species in KmerFinder: result from species identification using the KmerFinder database; MLST: sequence type; GC%: genome GC content; Contigs: number of contigs in genome; Size: genome size in Mbp; Weight of fish: weight of the fish from which the strain was isolated; Year: isolation year; Province: province from which the strain was isolated.

Isolate	PCR	Species in KmerFinder	MLST	GC%	Contigs	Size (Mbp)	Weight of Fish (g)	Year	Province
EI02	*E. ictaluri*	*E. ictaluri*	26	57.4	209	3.62	20	2017	Dong Thap
EI04	*E. ictaluri*	*E. ictaluri*	26	57.1	233	3.75	20	2017	Dong Thap
EI08	*E. ictaluri*	*E. ictaluri*	26	57.3	219	3.74	5	2017	An Giang
EI12	*E. ictaluri*	*E. ictaluri*	26	57.1	231	3.70	100	2017	Can Tho
EI14	*E. ictaluri*	*E. ictaluri*	26	57.4	190	3.61	100	2017	Can Tho
EI16	*E. ictaluri*	*E. ictaluri*	26	57.4	196	3.67	20	2018	Tien Giang
EI17	*E. ictaluri*	*E. ictaluri*	26	57.4	200	3.67	30	2018	Tien Giang
EI18	*E. ictaluri*	*E. ictaluri*	26	57.0	219	3.74	40	2018	Dong Thap
EI19	*E. ictaluri*	*E. ictaluri*	26	57.1	226	3.82	40	2018	Dong Thap
EI20	*E. ictaluri*	*E. ictaluri*	26	57.3	230	3.74	100	2019	Can Tho
EI21	*E. ictaluri*	*E. ictaluri*	26	57.3	214	3.75	75	2019	Tien Giang
EI24	*E. ictaluri*	*E. ictaluri*	26	57.4	202	3.55	75	2019	Can Tho
EI26	*E. ictaluri*	*E. ictaluri*	26	57.4	201	3.66	55	2019	Vinh Long
EI37	*E. ictaluri*	*E. ictaluri*	26	57.6	183	3.63	50	2019	Can Tho
EI41	*E. ictaluri*	*E. ictaluri*	26	57.7	195	3.56	40	2019	An Giang
EI44	*E. ictaluri*	*E. ictaluri*	26	57.3	208	3.74	35	2019	Ben Tre
EI48	*E. ictaluri*	*E. ictaluri*	26	57.1	190	3.75	30	2019	Dong Thap
EI51	*E. ictaluri*	*E. ictaluri*	26	57.1	214	3.75	15	2020	An Giang
EI52	*E. ictaluri*	*E. ictaluri*	26	57.3	195	3.74	100	2020	Dong Thap
EI53	*E. ictaluri*	*E. ictaluri*	26	57.4	222	3.63	750	2020	An Giang
EI54	*E. ictaluri*	*E. ictaluri*	26	57.4	201	3.65	25	2020	Can Tho
EI55	*E. ictaluri*	*E. ictaluri*	26	57.4	183	3.63	520	2020	Dong Thap
EI58	*E. ictaluri*	*E. ictaluri*	26	57.4	185	3.62	250	2020	Dong Thap
EI59	*E. ictaluri*	*E. ictaluri*	26	57.3	193	3.74	50	2021	Dong Thap
EI60	*E. ictaluri*	*E. ictaluri*	26	57.7	186	3.56	800	2021	Dong Thap
EI62	*E. ictaluri*	*E. ictaluri*	26	57.4	188	3.67	400	2021	Dong Thap
EI65	*E. ictaluri*	*E. ictaluri*	26	57.4	183	3.67	180	2021	Ben Tre
EI67	*E. ictaluri*	*E. ictaluri*	26	57.3	192	3.70	45	2021	Ben Tre
EI71	*E. ictaluri*	*E. ictaluri*	26	57.4	183	3.55	60	2021	Can Tho
EI72	*E. ictaluri*	*E. ictaluri*	26	57.4	203	3.66	25	2021	Long An

**Table 2:** Summary of plasmids found in the sequenced *E. ictaluri* isolates.

Isolate	Plasmid	Coverage (%)	Identity (%)	Plasmid Accession
EI04	IncA/C2	100	92.1	JN157804
EI12	IncA/C2	100	92.1	JN157804
	p0111	100	98.5	AP010962
EI18	IncA/C2	100	92.1	JN157804
	p0111	100	98.5	AP010962
EI19	IncA/C2	100	92.1	JN157804
EI48	p0111	100	98.5	AP010962
	IncA/C2	100	92.1	JN157804
	IncQ1	93.9	89.9	M28829.1
EI51	IncA/C2	100	92.1	JN157804
	p0111	100	98.5	AP010962

## Data Availability

The original data presented in the study are openly available in European Nucleotide Archive (ENA) under the designated project number PRJEB73297. The virulence gene database file is accessible as a text file in figshare through the public DOI: 10.6084/m9.figshare.25334749.

## Supplementary Materials

The following supporting information can be downloaded at: https://www.mdpi.com/article/10.3390/microorganisms12061182/s1, Table S1: NCBI sequence metadata, Table S2: SNP matrix, Table S3: Prophage results, and Table S4: Mobile Genetic Elements Results.
